# Clinical outcomes of COVID-19 in patients with sickle cell disease in French Guiana

**DOI:** 10.1016/j.heliyon.2024.e32017

**Published:** 2024-05-31

**Authors:** Narcisse Elenga, Baltazar Ntab, Joddy Mafema Missindu, Noelis Thomas Boizan, Alio Abassi

**Affiliations:** a Service de Pédiatrie, Centre Hospitalier de Cayenne, Rue des Flamboyants, BP 6006, 97306 Cayenne, Cedex, Guiana, France; b Département de L’Information Médicale, Centre Hospitalier Franck Joly 1465 Boulevard de La Liberté, BP 245, 97393, Saint-Laurent-du-Maroni, Guiana, France; c Centre de Reference de La Drépanocytose, Centre Hospitalier de Cayenne, Rue des Flamboyants, BP 6006, 97306, Cayenne, Cedex, Guiana, France

**Keywords:** Sickle cell disease, SARS-CoV2 infection, Favorable outcome, French Guiana

## Abstract

**Objectives:**

French Guiana also suffered concrete epidemiological consequences of the SARS-CoV2 epidemic. These regions are home to a large number of sickle cell patients who are at risk of developing severe forms of COVID-19. This study aimed to describe the characteristics and prognosis of patients with sickle disease infected with SARS-CoV2 during the first and second epidemic waves of 2020 in French Guiana.

**Design:**

We conducted a multicenter, retrospective cohort study that included sickle cell patients followed up in French Guiana.

**Results:**

Among the 79 patients infected with SARS-CoV2, there were 26 children <18 years. Forty-two patients were hospitalized and 37 were outpatients. Respiratory illness (38 %), vaso-occlusive crises (18 %), and acute chest syndrome (8 %) were the main causes of hospitalization. There were two cases of pulmonary embolism and one case of deep limb thrombosis. Seven patients were transferred to the ICU. Of these, three received mechanical ventilation, and two died. The deceased patients had the SS phenotype (a 16-year-old boy and a 32-year-old man), both of whom exhibited severe pneumonitis complicated by COVID-19.

**Conclusion:**

Despite the two deaths, the overall outcome was favorable.

## Highlights

• Overall outcomes were favorable.
• The manifestation of COVID-19 in our study population was mild to moderate.
• Patients with the SC genotype also had a favorable outcome.

## 1 Introduction

The global pandemic of severe acute respiratory syndrome coronavirus 2 (SARS-CoV-2) began in December 2009 in Wuhan, China [1]. Since then, several publications have focused on the future of patients with sickle cell disease (SCD). According to these studies, COVID-19 appears to pose a threat to people with SCD, and the risk is particularly high in individuals with the SC genotype [2]. In French Guiana, where the incidence of SCD is high and the prevalence of SC disease is 35 %, there is a risk if we consider the data in the literature. Due to endothelial dysfunction and chronic inflammation, patients with SCD are at risk of complications such as vaso-occlusive crises during febrile episodes. These episodes may be exacerbated by acute chest syndrome, particularly in the presence of pulmonary involvement, as in COVID-19 disease. Microvascular ischaemia, endothelial activation and inflammation due to cytokine release play an important role in the pathogenesis of SCD. However, COVID-19 acts through the angiotensin 2 converting enzyme, CD147 and CD26 in red blood cells and can lead to hemoglobin dysfunction and reduced oxygen-carrying capacity of hemoglobin [3]. It can also cause vaso-occlusive coagulopathy, increased vascular permeability, and fluid accumulation in the lungs, leading to potentially fatal complications of sickle cell disease. This study aimed to describe the characteristics and prognosis of SCD infected with SARS-CoV2 during the first and second epidemic waves of 2020 in French Guiana, where the prevalence of SCD is relatively high.

## 2 Materials and methods

### 2.1 Organizations during the COVID-19 epidemic

During the COVID-19 pandemic, sickle cell patients were confined to their homes and ordered not to go to the hospital. All scheduled consultations at the reference centers were cancelled. Information was provided by telephone to the patient: “telephone consultation, confinement instructions, stay at home, telework or sick leave, social distancing, prevention barriers, continuation of treatment, avoid non-steroidal anti-inflammatory drugs, get vaccinated.” Repeated information on the warning signs of COVID-19 was systematically provided to patients. The patients only attended the hospital when they exhibited clinical signs of the disease. Despite a relatively early start and considerable efforts by local and national health authorities to communicate and reach the most socially isolated populations by April 2022, only 29.8 % of the population has completed vaccination [4].

### 2.2 Study design and participants

In this multicenter retrospective study, we included all children and adult sickle cell patients followed up at the Cayenne Sickle Cell Center and the Franck Joly Hospital in Saint-Laurent-du-Maroni, with symptoms suggestive of COVD-19 and a positive test for SARS-CoV-2. Data collected from the patient records were entered into an Excel file for analysis. These data included demographic data; clinical signs such as fever, digestive disturbances, and respiratory signs before, during hospitalization, and during follow-up after discharge; blood count; CRP and creatinine levels; and microbiological results (PCR-SARS-CoV-2 test).

### 2.3 COVID-19 testing procedures

The diagnosis was confirmed by a positive SARS-COV2 reverse transcription polymerase chain reaction (RT-PCR) test on a nasopharyngeal swab.

### 2.4 Statistical analysis

All analyses were performed using STATA 15.0 software (StataCorp LP, College Station, TX, USA). Quantitative variables were statistically classified using the first and third quartiles and median. We performed simple comparisons using the Student's t-test for quantitative variables and CHI2 or Fisher's exact test for qualitative variables. Bivariate analysis was performed to examine the covariates and their relationships with outcome measures based on the crude odds ratio and its confidence interval. The results were expressed as means, odds ratios, and confidence intervals.

### 2.5 Ethical approval

This study was based on existing healthcare data. These data were extracted from hospital databases and anonymously collected. In addition, the sickle cell patient database has already been declared by the Commission Nationale Informatique et Liberté (CNIL, number 2200250 v 0). The patients were informed of the use of their data by an information poster in the medical units concerned with SCD. All patients were personally informed and asked to provide consent to participate in the study. All data were collected after certification of the patients’ written non-opposition. All participants who were minors provided written informed consent from their parents or legal guardians.

## 3 Results

We enrolled 79 patients infected with SARS-CoV2. Of these, 26 were under 18 years of age. The sociodemographic characteristics of the patients are summarized in Table 1. There were no statistically significant differences in demographic, clinical, and comorbidity parameters between patients with different genotypes. The two groups were also comparable in terms of clinical and biological course, except for neutrophil and CRP levels (Table 2). COVID-19 vaccination completed in 30.4 % of the study population (Table 2). Forty-two patients were hospitalized and 37 were outpatients. Respiratory diseases, followed by vaso-occlusive crises (18 %) and acute chest syndrome (8 %), were the main causes of hospitalization. There were two cases of pulmonary embolism and one case of deep limb thrombosis. Seven patients were assigned to the IUC group. Of these, three received mechanical ventilation. Unvaccinated patients more likely to receive mechanical ventilation (3–0, p < 0.001). The two patients who died had the SS phenotype (a 16-year-old boy and a 32-year-old man), both of whom presented with severe COVID-19 pneumonitis complicated by acute chest syndrome and pulmonary embolism. The two patients who died had not been vaccinated. The clinical results are summarized in Table 2 and Table 3.

**Table 1:** Demographic and clinical characteristics and basal hemoglobin levels of the sickle cell patients infected with SARS‐CoV‐2 in French Guiana.

**Variables/N**		Cohort	SC/Sβ+	SS/Sβ°	**p**
		**79**	**11/9**	**59/0**	
Age	6	22[14–2]	25 [20–33]	21[12–31]	0.05
Sex	M/F	40/39	8/12	28/31	0.3
BMI	Median [Q25—Q75]	21.4[18.5–24.5]	22.5[19.5–25.5]	21.5[18.5–23.5]	0.2
Basal Hb (g/dL)	Median [Q25—Q75]	8.6[7.5–11.5]	9.5[8.5–11.5]	8.4[7.5–9.5]	0.3
**Comorbid conditions**					
Chronic kidney disease	n (%)	0(0)	0 (0)	0 (0)	–
Renal insufficiency	n (%)	0(0)	0 (0)	0 (0)	–
Cardiac insufficiency	n (%)	0(0)	0 (0)	0 (0)	–
Obstructive pulmonary disease	n (%)	0(0)	0 (0)	0 (0)	–
Cerebral vasculopathy	n (%)	2 (2.54)	1 (1.27)	1 (1.27)	0.4
>1 ACS	n (%)	22 (28)	4 (5)	18 (23)	0.4
Vaccinated to COVID-19	n (%)	24 (30)	4 (20)	20 (34)	0.2
Current treatment					
Hydroxyurea	n (%)	27 (34)	5 (6)	22(28)	0.3
Exchange transfusion program	n (%)	0 (0.0)	0 (0.0)	0 (0.0)	–
Transfusion's program	n (%)	0 (0.0)	0 (0.0)	0 (0.0)	–
Phlebotomy's program	n (%)	0 (0.0)	0 (0.0)	0 (0.0)	–
ACE inhibitor	n (%)	0 (0.0)	0 (0.0)	0 (0.0)	–
Home oxygen therapy	n (%)	0 (0.0)	0 (0.0)	0 (0.0)	–

Abbreviations: ACS, acute chest syndrome; ACE inhibitor, angiotensin‐converting enzyme inhibitor; BMI, body mass index; Hb, hemoglobin; VOC, vaso‐occlusive crisis. *p*‐Values compare SC/Sβ+ with SS/Sβ0 patients. Bold *p*‐values indicate statistically significant results (*p* < 0.05).

**Table 2:** Biological outcomes of the sickle cell patients infected with SARS‐CoV‐2 in French Guiana.

Genotyes	SC/Sβ+	SS/Sβ°	p
	11/9	59/0	
**Biological outcomes (Median [Q25–Q75])**			
Lowest value of Hb (g/dl)	9.4 [7.8–10.5]	8.6 [6.7–9.9]	0.323
Highest value of neutrophils (G/L)	3.7 [3.0–5.9]	8.3 [5.6–11.1]	0.003
Lowest value of lymphocytes (G/L)	2.0 [1.5–3.2]	1.7 [0.8–2.7]	0.275
Highest value of creatinine (μmol/l)	56.5 [53.5–64.5]	60.5 [45.0–72.3]	0.853
Highest value of CRP (mg/L)	11.0 [4.9–17.3]	71.0 [40.0–202.0]	0.005

**Table 3:** Biological outcomes of the sickle cell patients infected with SARS‐CoV‐2 in French Guiana.

Comorbid conditions N(%)	Outpatients	Inpatients	p
**Biological outcomes (Median [Q25–Q75])**			
Lowest value of Hb (g/dl)	9.4 [7.8–10.5]	8.6 [6.7–9.9]	0.323
Highest value of neutrophils (G/L)	3.7 [3.0–5.9]	8.3 [5.6–11.1]	**0.003**
Lowest value of lymphocytes (G/L)	2.0 [1.5–3.2]	1.7 [0.8–2.7]	0.275
Highest value of creatinine (μmol/l)	56.5 [53.5–64.5]	60.5 [45.0–72.3]	0.853
Highest value of CRP (mg/L)	11.0 [4.9–17.3]	71.0 [40.0–202.0]	**0.005**

## 4 Discussion

The manifestation of COVID-19 in our study population was mild to moderate in most pediatric and adult patients, and even in patients with the SC genotype. Our study showed a low incidence of COVID-19 and a milder disease spectrum in the study population. These results suggest that there is no increased risk of mortality or morbidity associated with COVID-19 in patients with sickle cell disease. It is certainly true that our study population was young with few comorbidities; however, the rapid and coordinated management of patients at the reference center could also explain this favorable outcome. In addition, the prophylactic use of sickle cell disease-modifying therapies, such as hydroxyurea, has been suggested as a possible cause of positive COVID-19 results in patients with SCD [5]. The possible benefits of HU include increased HbF, reduced hemolysis, and reduced activation of platelets, leukocytes, and inflammatory parameters such as interleukin. Other studies have shown similar results [6,7]. Some authors have suggested that sickle cell disease may provide a biological advantage against SARS-CoV-2 infection in the same way that sickle cell disease protects against malaria. Indeed, the characteristic state of chronic inflammation and hemolytic anemia associated with the disease may protect individuals against the severe presentations of COVID-19 [8]. However, further studies are required to confirm these hypotheses. However, these results should be interpreted with caution. Indeed, several studies have suggested that sickle cell disease confers an increased risk of severe COVID-19, COVID-19 triggering sickle cell complications [[9], [10], [11]]. According to these studies, patients with SCD are at risk of severe forms of the disease, with many hospitalizations in intensive care units and many more deaths, especially for the SC genotype.

During an infectious process such as SARS-COV2 infection, the chronic inflammatory state associated with sickle cell disease is exacerbated. Leukocyte recruitment, cytokine release, and direct damage to the endothelium further increase hypercoagulability, with enhanced vasoconstriction, platelet stimulation, and adhesion molecule secretion, favoring the onset of vaso-occlusion or acute chest syndrome. In conclusion, an analysis of the data available in the literature on the effect of COVID-19 on people with SCD revealed many discrepancies [11]. Rather than defining people with SCD as belonging to a risk group, the international scientific community should focus on understanding the specificities of the interaction between COVID-19 and SCD, and the clinical course of SARS-CoV-2 infection in the context of SCD. Furthermore, the reason for the low vaccination coverage against COVID-19 in our study could be the fear of unpredictable complications from the COVID vaccine. Studies have shown that people with SCD may have concerns about the safety profile of new vaccines and the occurrence of adverse reactions [12]. Vaccination is the best way to reduce infectious mortality in the sickle cell population, and should be widely promoted. To achieve this, health professionals must dispel myths and highlight the proven benefits of vaccination.

Although our study reported favorable outcomes for patients with SCD and COVID-19, the long-term effects of SARS-CoV-2 infection are unknown and may affect individuals with SCD differently than in the general population. Future research should focus on multicenter studies with larger sample sizes, evaluation of SCD-modifying therapies on COVID-19 outcomes, inclusion of case-matched controls that account for the unique characteristics of SCD populations, and longitudinal assessment of symptoms after COVID-19.

### 4.1 Study limitations

The limitations of our study include the lack of data on organic complications, socioeconomic factors, and biological or radiological findings during hospitalization. Another limitation related to the retrospective nature of our study was the size of the sample.

## 5 Conclusion

In conclusion, our study shows a favorable evolution for patients with SCD affected by covid-19, although there are many discrepancies in the literature. Vaccination coverage among patients with sickle cells should be improved.

## Funding

This research received no specific grants from any funding agency in the public, commercial, or not-for-profit sector.

## Data availability

Data will be made available on request.

## CRediT authorship contribution statement

**Narcisse Elenga:** Writing – review & editing, Writing – original draft, Validation, Supervision, Methodology, Investigation, Formal analysis, Data curation, Conceptualization. **Baltazar Ntab:** Validation, Resources, Data curation. **Joddy Mafema Missindu:** Validation, Resources, Data curation. **Noelis Thomas Boizan:** Validation, Resources, Data curation. **Alio Abassi:** Writing – review & editing, Formal analysis, Data curation, Conceptualization.

## Declaration of competing interest

The authors declare that they have no competing interests.
